# 1391. "Going Viral for Good: The Global Impact of #IDTwitter in the Infectious Diseases Twitter Community"

**DOI:** 10.1093/ofid/ofad500.1228

**Published:** 2023-11-27

**Authors:** Priyal Mehta, Smitesh S Padte, Diksha Mahendru, Sawsan Tawfeeq, Atanas Atanasov, Zara Arshad, Rahul Kashyap, Faisal A Nawaz

**Affiliations:** MW Desai General Municipal Hospital, Mumbai, Maharashtra, India; MW Desai General Municipal Hospital, Mumbai, Maharashtra, India; Global Remote Researchers Scholars Program, Ludhiana, Punjab, India; Global Remote Researchers Scholars Program, Ludhiana, Punjab, India; Ludwig Boltzmann and Patient Safety, Medical University of Vienna, Vienna, Wien, Austria; Shifa International Hospital, Islamabad, Islamabad, Pakistan; Mayo Clinic, Rochester, Minnesota; Department of Psychiatry, Al Amal Psychiatric Hospital, Dubai, United Arab Emirates, Dubai, Dubai, United Arab Emirates

## Abstract

**Background:**

Twitter has become an invaluable resource for gaining insights into crucial developments in global healthcare communication. The hashtags (#) can be used to categorize tweets and gather conversations on a specific topic or to target a particular audience. Although the prevailing knowledge fund underlines the potential of digital networks to essentially influence the management of infectious diseases (ID), there has been no comprehensive analysis on the user information in the ID Twitter community, nor of the influence this hashtag has generated. The objective of our study was to evaluate the demographic data of #IDTwitter users, discern the most influential members and popular narratives in this realm, and ascertain the impact of the hashtag.

**Methods:**

Using data from 28th June 2019 and 28th March 2023, an extensive analysis was conducted using the Symplur Signals research analytics tool. The analysis focused on the cumulative number of tweets, impressions, and unique users who shared tweets containing the hashtag #IDTwitter, with users categorized into specific healthcare stakeholder groups. The primary outcome measures were outreach and awareness measured by the number of tweets and impressions.

**Results:**

The study observed the trends of #IDTwitter over a period of 45 months and found 441,650 tweets were shared by 92,734 users that generated a total of 1,833,037,732 impressions (views). Top five co-occurring hashtags were #IDtwitter, #MedTwitter, #MedEd, #COVID19, #TwitteRx. The top five countries reporting the greatest number of users of this hashtag were The United States of America (48326), Canada (4803), Mexico (3413), India (2759), Australia (2658). Various healthcare stakeholders’ categories were identified and three largest groups of contributors were Doctors (14.55%), Healthcare Providers (7.54%) and Researcher/Academic (3.70%). The top three influencers of this hashtag include two clinical pharmacists and one organization account.

Country-wise distribution of Users of #IDTwitter

**Figure:**
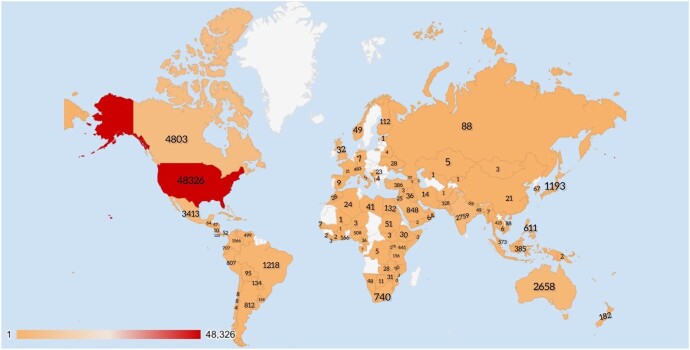

The image shows the geographical distribution of the users who posted tweets containing #IDTwitter were shared (based on the locations at which the posting accounts were registered). Twitter is used worldwide for conversations regarding communicable diseases and their prevention, not only among the general public but also among students and professionals via online chat discussions and virtual rounds.

Stakeholders of #IDTwitter

**Figure:**
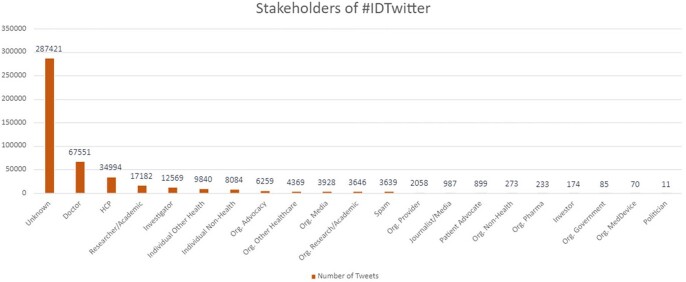

Accounting for the percentage distribution of #IDTwitter-posting users in various healthcare stakeholders categories (data derived from Symplur Signals, with the classification being based on information provided in the Twitter biographies of the users- https://help.symplur.com/en/articles/103684-healthcare-stakeholder-segmentation). Twitter has become a quintessential tool for connecting people worldwide, and we can leverage this platform to our advantage by paying close attention to the topic and content of hashtag exchanges to combat misinformation related to matters like antibiotic usage or any epidemic infection on social media platforms.

Top Co-occurring Hashtags

**Figure:**
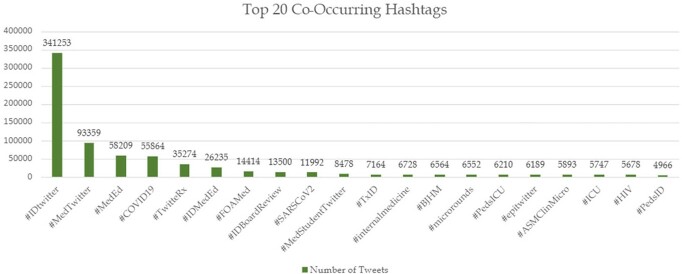

The hashtags that generally appear alongside of #IDTwitter could provide an insight regarding the popular discussions involving #IDTwitter.

**Conclusion:**

Our findings indicate that there is considerable interest in using #IDTwitter to promote relevant content and engage a geographically diverse audience. It underscores the vitality of professional voices in combating misinformation and we could definitely leverage this 'viral' hashtag for our advantage.

**Disclosures:**

**All Authors**: No reported disclosures

